# (7-Dimethylamino-1-hydroxy-3-naphthyl)(morpholino)methanone

**DOI:** 10.1107/S1600536809051848

**Published:** 2009-12-09

**Authors:** Moon-Hwan Kim, Ji-Su Seo, Chong-Hyeak Kim, Jae-Wook Ryu, Ki-Hwan Lee

**Affiliations:** a Biomaterial Research Center, Korea Research Institute of Chemical Technology, PO Box 107, Yuseong, Daejeon 305-600, Republic of Korea; b Center for Chemical Analysis, Korea Research Institute of Chemical Technology, PO Box 107, Yuseong, Daejeon 305-600, Republic of Korea; c Department of Chemistry, Kongju National University, Kongju 314-701, Republic of Korea

## Abstract

In the title compound, C_17_H_20_N_2_O_3_, the morpholine ring is in a slightly distorted chair form. The crystal structure is stabilized by an inter­molecular O—H⋯O hydrogen bond between the H atom of the hydroxyl group and the O atom of a neighbouring carbonyl group. A weak inter­molecular C—H⋯π inter­action is also present.

## Related literature

For the synthesis and applications of organic photochromic dyes, see: Gabbutt *et al.* (2003 ▶, 2004 ▶); Kumar *et al.* (1995 ▶); Gemert & Selvig (2000 ▶); Nelson *et al.* (2002 ▶). For their potential use as variable optical transmission materials and in optical storage, see; Crano & Guglielmetti (1999 ▶).
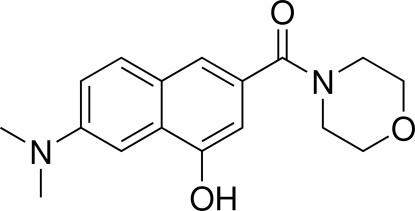


## Experimental

### 

#### Crystal data

C_17_H_20_N_2_O_3_
*M* *_r_* = 300.35
Orthorhombic, 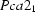

*a* = 12.6250 (5) Å
*b* = 13.9634 (6) Å
*c* = 8.8369 (3) Å
*V* = 1557.84 (11) Å^3^
*Z* = 4
Mo *K*α radiation
μ = 0.09 mm^−1^
*T* = 296 K
0.41 × 0.18 × 0.08 mm

#### Data collection

Bruker APEXII CCD diffractometer
8069 measured reflections
2044 independent reflections
1475 reflections with *I* > 2σ(*I*)
*R*_int_ = 0.025

#### Refinement

*R*[*F* ^2^ > 2σ(*F* ^2^)] = 0.044
*wR*(*F* ^2^) = 0.115
*S* = 1.04
2044 reflections
200 parameters
1 restraint
H-atom parameters constrained
Δρ_max_ = 0.19 e Å^−3^
Δρ_min_ = −0.15 e Å^−3^

### 

Data collection: *APEX2* (Bruker, 2007 ▶); cell refinement: *SAINT* (Bruker, 2007 ▶); data reduction: *SAINT*; program(s) used to solve structure: *SHELXS97* (Sheldrick, 2008 ▶); program(s) used to refine structure: *SHELXL97* (Sheldrick, 2008 ▶); molecular graphics: *SHELXTL* (Sheldrick, 2008 ▶); software used to prepare material for publication: *SHELXTL*.

## Supplementary Material

Crystal structure: contains datablocks global, I. DOI: 10.1107/S1600536809051848/lx2129sup1.cif

Structure factors: contains datablocks I. DOI: 10.1107/S1600536809051848/lx2129Isup2.hkl

Additional supplementary materials:  crystallographic information; 3D view; checkCIF report

## Figures and Tables

**Table 1:** Hydrogen-bond geometry (Å, °)

*D*—H⋯*A*	*D*—H	H⋯*A*	*D*⋯*A*	*D*—H⋯*A*
O1—H1⋯O2^i^	0.82	1.82	2.631 (3)	172
C17—H17*C*⋯*Cg*^ii^	0.96	2.80	3.533 (2)	134

Symmetry codes: (i) 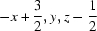; (ii) 
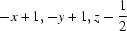
. *Cg* is the centroid of the C5–C10 benzene ring.

**Fig. 1.**
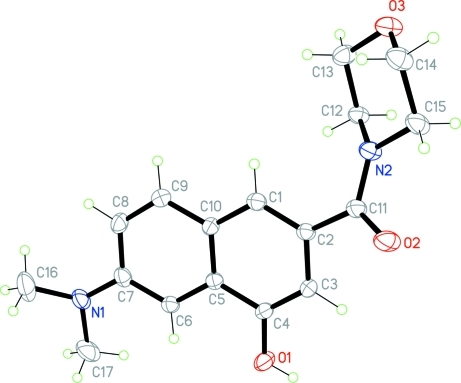
The molecular structure of the title compound with the atom numbering scheme. Displacement ellipsoids are drawn at the 30% probability level. H atoms are presented as a small spheres of arbitrary radius.

**Fig. 2.**
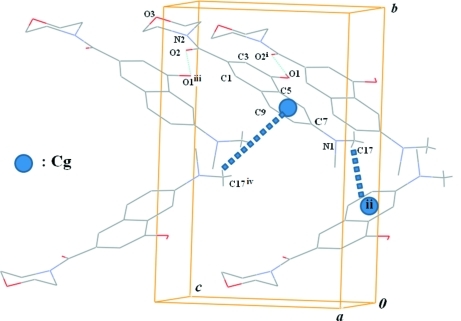
O—H···O and C—H···π interactions (dotted lines) in the crystal structure of the title compound. Cg denotes the ring centroid. [Symmetry codes: (i) - x + 3/2, y, z -1/2; (ii) - x + 1, - y + 1, z - 1/2; (iii) - x + 3/2, y, z + 1/2; (iv) - x + 1, - y + 1, z + 1/2.]

## supplementary crystallographic information

### Comment

The synthesis of organic photochromic dyes and their application has become of great interest recently (Gabbutt *et al.*, 2003, 2004; Kumar *et al.*, 1995; Gemert & Selvig, 2000; Nelson *et al.*, 2002. Because they may be useful such as variable optical transmission materials (ophthalmic glasses and lenses) or in potential use such as optical storage (optical disks or memories) (Crano & Guglielmetti, 1999). Here we report the crystal structure of the title compound (Fig. 1). In the title compound, the conformation of the morpholine ring is in a slightly distorted chair form. The crystal packing (Fig. 2) is stabilized by an intermolecular O—H···O hydrogen bond between the H atom of the hydroxyl group and the O atom of a neighbouring C═O unit, with a O1—H1···O2^i^ (Table 1). The molecular packing (Fig. 2) is further stabilized by a intermolecular C—H···π interaction between a methyl H atom of the dimethylamino group and the N-bonded benzene ring, with a C17—H17C···Cg^ii^ (Table 1; Cg is the centroid of the C5–C10 benzene ring).

### Experimental

The title compound was synthesized from the reaction of 1-hydroxy-7-dimethylamino-3-naphthonic acid (116 g, 0.5 mol) and morpholine (48 g, 1.2 mol) in anhydrous CH_2_Cl_2_ for 24 h at room temperature. The reaction was quenched by the addition of water and the organic layer separated, dried over anhydrous MgSO_4_, filtered and concentrated to give the title compound (120 g, yield 81%). Single crystals suitable for X-ray diffraction were prepared by evaporation of a solution of the title compound in ethyl acetate at room temperature.

### Refinement

All the Friedel pairs were merged. All hydrogen atoms were placed in calculated positions using a riding model, with C—H = 0.93–0.97 Å, O—H = 0.82 Å, and with *U*_iso_(H) = 1.2–1.5 *U*_eq_(C, O).

### Crystal data

**Table:**

C_17_H_20_N_2_O_3_	*F*(000) = 640
*M**_r_* = 300.35	*D*_x_ = 1.281 Mg m^−^^3^
Orthorhombic, *P**c**a*2_1_	Mo *K*α radiation, λ = 0.71073 Å
Hall symbol: P 2c -2ac	Cell parameters from 2694 reflections
*a* = 12.6250 (5) Å	θ = 2.9–22.0°
*b* = 13.9634 (6) Å	µ = 0.09 mm^−^^1^
*c* = 8.8369 (3) Å	*T* = 296 K
*V* = 1557.84 (11) Å^3^	Block, silver
*Z* = 4	0.41 × 0.18 × 0.08 mm

### Data collection

**Table:**

Bruker APEXII CCD diffractometer	1475 reflections with *I* > 2σ(*I*)
Radiation source: fine-focus sealed tube	*R*_int_ = 0.025
graphite	θ_max_ = 28.3°, θ_min_ = 1.5°
Detector resolution: 10 pixels mm^-1^	*h* = −16→11
φ and ω scans	*k* = −9→18
8069 measured reflections	*l* = −9→11
2044 independent reflections	

### Refinement

**Table:**

Refinement on *F*^2^	Secondary atom site location: difference Fourier map
Least-squares matrix: full	Hydrogen site location: difference Fourier map
*R*[*F*^2^ > 2σ(*F*^2^)] = 0.044	H-atom parameters constrained
*wR*(*F*^2^) = 0.115	*w* = 1/[σ^2^(*F*_o_^2^) + (0.0628*P*)^2^ + 0.0129*P*] where *P* = (*F*_o_^2^ + 2*F*_c_^2^)/3
*S* = 1.04	(Δ/σ)_max_ < 0.001
2044 reflections	Δρ_max_ = 0.19 e Å^−^^3^
200 parameters	Δρ_min_ = −0.15 e Å^−^^3^
1 restraint	Extinction correction: *SHELXL*, Fc^*^=kFc[1+0.001xFc^2^λ^3^/sin(2θ)]^-1/4^
Primary atom site location: structure-invariant direct methods	Extinction coefficient: 0.009 (2)

### Special details

**Table:**

Geometry. All esds (except the esd in the dihedral angle between two l.s. planes) are estimated using the full covariance matrix. The cell esds are taken into account individually in the estimation of esds in distances, angles and torsion angles; correlations between esds in cell parameters are only used when they are defined by crystal symmetry. An approximate (isotropic) treatment of cell esds is used for estimating esds involving l.s. planes.
Refinement. Refinement of F^2^ against ALL reflections. The weighted R-factor wR and goodness of fit S are based on F^2^, conventional R-factors R are based on F, with F set to zero for negative F^2^. The threshold expression of F^2^ > 2sigma(F^2^) is used only for calculating R-factors(gt) etc. and is not relevant to the choice of reflections for refinement. R-factors based on F^2^ are statistically about twice as large as those based on F, and R- factors based on ALL data will be even larger.

### Fractional atomic coordinates and isotropic or equivalent isotropic displacement parameters (Å^2^)

**Table:**

	*x*	*y*	*z*	*U*_iso_*/*U*_eq_	
O1	0.67137 (15)	0.76538 (16)	0.3748 (2)	0.0597 (6)	
H1	0.7257	0.7921	0.4033	0.090*	
O2	0.64658 (15)	0.84718 (18)	0.9402 (3)	0.0682 (6)	
O3	0.31381 (16)	0.94813 (16)	1.1678 (2)	0.0583 (6)	
N1	0.38830 (19)	0.55244 (19)	0.1665 (3)	0.0600 (8)	
N2	0.47850 (17)	0.89337 (17)	0.9690 (3)	0.0475 (6)	
C1	0.4410 (2)	0.76093 (18)	0.7082 (3)	0.0432 (6)	
H1A	0.3886	0.7595	0.7821	0.052*	
C2	0.53284 (19)	0.81050 (16)	0.7348 (3)	0.0387 (6)	
C3	0.6131 (2)	0.81177 (19)	0.6231 (3)	0.0397 (6)	
H3A	0.6762	0.8439	0.6422	0.048*	
C4	0.59920 (19)	0.76642 (18)	0.4873 (3)	0.0389 (6)	
C5	0.50466 (19)	0.71367 (16)	0.4583 (3)	0.0362 (5)	
C6	0.4919 (2)	0.66117 (17)	0.3236 (3)	0.0412 (6)	
H6A	0.5446	0.6634	0.2501	0.049*	
C7	0.4022 (2)	0.60611 (19)	0.2982 (3)	0.0465 (7)	
C8	0.3216 (2)	0.6086 (2)	0.4082 (4)	0.0514 (8)	
H8A	0.2593	0.5746	0.3911	0.062*	
C9	0.3321 (2)	0.65914 (19)	0.5389 (4)	0.0483 (7)	
H9A	0.2772	0.6589	0.6090	0.058*	
C10	0.4249 (2)	0.71200 (17)	0.5702 (3)	0.0383 (6)	
C11	0.5567 (2)	0.8524 (2)	0.8856 (3)	0.0448 (6)	
C12	0.38259 (19)	0.94019 (19)	0.9127 (3)	0.0429 (6)	
H12A	0.3663	0.9165	0.8121	0.051*	
H12B	0.3944	1.0087	0.9058	0.051*	
C13	0.2917 (2)	0.9210 (2)	1.0154 (4)	0.0552 (8)	
H13A	0.2303	0.9561	0.9799	0.066*	
H13B	0.2748	0.8533	1.0124	0.066*	
C14	0.4045 (2)	0.8996 (2)	1.2216 (4)	0.0616 (8)	
H14A	0.3910	0.8312	1.2215	0.074*	
H14B	0.4184	0.9190	1.3251	0.074*	
C15	0.5006 (2)	0.9204 (2)	1.1257 (3)	0.0595 (9)	
H15A	0.5177	0.9880	1.1310	0.071*	
H15B	0.5608	0.8844	1.1631	0.071*	
C16	0.3346 (3)	0.4603 (2)	0.1784 (5)	0.0888 (13)	
H16A	0.2810	0.4639	0.2552	0.133*	
H16B	0.3850	0.4116	0.2047	0.133*	
H16C	0.3024	0.4448	0.0831	0.133*	
C17	0.4705 (3)	0.5571 (3)	0.0525 (5)	0.0855 (13)	
H17A	0.5021	0.6196	0.0533	0.128*	
H17B	0.4402	0.5449	−0.0453	0.128*	
H17C	0.5236	0.5098	0.0738	0.128*	

### Atomic displacement parameters (Å^2^)

**Table:**

	*U*^11^	*U*^22^	*U*^33^	*U*^12^	*U*^13^	*U*^23^
O1	0.0421 (11)	0.0899 (15)	0.0471 (12)	−0.0209 (10)	0.0090 (10)	−0.0154 (11)
O2	0.0366 (11)	0.1086 (17)	0.0593 (13)	0.0192 (11)	−0.0110 (11)	−0.0273 (12)
O3	0.0509 (12)	0.0789 (14)	0.0452 (11)	0.0120 (11)	0.0080 (9)	0.0015 (10)
N1	0.0498 (16)	0.0608 (15)	0.0693 (18)	0.0048 (12)	−0.0095 (14)	−0.0306 (13)
N2	0.0353 (12)	0.0696 (15)	0.0375 (12)	0.0122 (11)	−0.0063 (11)	−0.0107 (11)
C1	0.0382 (15)	0.0469 (14)	0.0443 (16)	0.0039 (12)	0.0031 (13)	−0.0048 (12)
C2	0.0342 (14)	0.0419 (13)	0.0400 (14)	0.0046 (11)	0.0011 (12)	−0.0038 (11)
C3	0.0314 (15)	0.0431 (14)	0.0446 (15)	−0.0028 (11)	−0.0034 (12)	−0.0046 (11)
C4	0.0312 (14)	0.0436 (13)	0.0419 (14)	−0.0025 (11)	0.0033 (12)	−0.0003 (12)
C5	0.0332 (13)	0.0360 (12)	0.0394 (14)	0.0048 (10)	−0.0043 (11)	0.0004 (11)
C6	0.0359 (15)	0.0431 (13)	0.0446 (14)	0.0041 (12)	0.0002 (12)	−0.0042 (11)
C7	0.0443 (17)	0.0410 (14)	0.0542 (17)	0.0081 (12)	−0.0123 (14)	−0.0087 (12)
C8	0.0376 (16)	0.0493 (15)	0.067 (2)	−0.0063 (12)	−0.0098 (15)	−0.0082 (14)
C9	0.0379 (16)	0.0511 (15)	0.0557 (17)	−0.0018 (13)	0.0043 (13)	−0.0048 (14)
C10	0.0350 (14)	0.0370 (12)	0.0428 (14)	−0.0022 (11)	−0.0006 (12)	−0.0015 (10)
C11	0.0350 (16)	0.0535 (15)	0.0460 (15)	0.0050 (12)	−0.0005 (13)	−0.0093 (13)
C12	0.0377 (15)	0.0492 (14)	0.0417 (14)	0.0086 (12)	0.0018 (12)	−0.0014 (12)
C13	0.0396 (17)	0.071 (2)	0.0551 (19)	0.0011 (14)	−0.0013 (14)	0.0017 (15)
C14	0.064 (2)	0.077 (2)	0.0447 (16)	0.0134 (17)	−0.0017 (16)	−0.0045 (16)
C15	0.0465 (18)	0.086 (2)	0.0455 (17)	0.0145 (17)	−0.0083 (14)	−0.0217 (17)
C16	0.099 (3)	0.0587 (18)	0.109 (3)	−0.0026 (19)	−0.028 (3)	−0.032 (2)
C17	0.077 (2)	0.106 (3)	0.073 (2)	0.011 (2)	−0.002 (2)	−0.053 (2)

### Geometric parameters (Å, °)

**Table:**

O1—C4	1.349 (3)	C6—H6A	0.9300
O1—H1	0.8200	C7—C8	1.409 (4)
O2—C11	1.235 (3)	C8—C9	1.360 (4)
O3—C14	1.413 (4)	C8—H8A	0.9300
O3—C13	1.426 (4)	C9—C10	1.411 (4)
N1—C7	1.395 (4)	C9—H9A	0.9300
N1—C17	1.448 (4)	C12—C13	1.487 (4)
N1—C16	1.458 (4)	C12—H12A	0.9700
N2—C11	1.359 (3)	C12—H12B	0.9700
N2—C15	1.461 (4)	C13—H13A	0.9700
N2—C12	1.463 (3)	C13—H13B	0.9700
C1—C2	1.370 (3)	C14—C15	1.508 (4)
C1—C10	1.413 (4)	C14—H14A	0.9700
C1—H1A	0.9300	C14—H14B	0.9700
C2—C3	1.414 (4)	C15—H15A	0.9700
C2—C11	1.487 (4)	C15—H15B	0.9700
C3—C4	1.368 (4)	C16—H16A	0.9600
C3—H3A	0.9300	C16—H16B	0.9600
C4—C5	1.426 (3)	C16—H16C	0.9600
C5—C6	1.407 (4)	C17—H17A	0.9600
C5—C10	1.412 (4)	C17—H17B	0.9600
C6—C7	1.387 (4)	C17—H17C	0.9600
			
C4—O1—H1	109.5	O2—C11—C2	120.9 (2)
C14—O3—C13	110.4 (2)	N2—C11—C2	120.3 (2)
C7—N1—C17	117.8 (2)	N2—C12—C13	110.5 (2)
C7—N1—C16	118.2 (3)	N2—C12—H12A	109.5
C17—N1—C16	115.0 (3)	C13—C12—H12A	109.5
C11—N2—C15	118.9 (2)	N2—C12—H12B	109.5
C11—N2—C12	127.2 (2)	C13—C12—H12B	109.5
C15—N2—C12	111.4 (2)	H12A—C12—H12B	108.1
C2—C1—C10	120.9 (3)	O3—C13—C12	112.2 (2)
C2—C1—H1A	119.5	O3—C13—H13A	109.2
C10—C1—H1A	119.5	C12—C13—H13A	109.2
C1—C2—C3	119.5 (2)	O3—C13—H13B	109.2
C1—C2—C11	121.6 (2)	C12—C13—H13B	109.2
C3—C2—C11	118.4 (2)	H13A—C13—H13B	107.9
C4—C3—C2	121.0 (2)	O3—C14—C15	111.7 (3)
C4—C3—H3A	119.5	O3—C14—H14A	109.3
C2—C3—H3A	119.5	C15—C14—H14A	109.3
O1—C4—C3	124.4 (2)	O3—C14—H14B	109.3
O1—C4—C5	115.3 (2)	C15—C14—H14B	109.3
C3—C4—C5	120.3 (2)	H14A—C14—H14B	107.9
C6—C5—C10	120.2 (2)	N2—C15—C14	109.2 (2)
C6—C5—C4	121.1 (2)	N2—C15—H15A	109.8
C10—C5—C4	118.7 (2)	C14—C15—H15A	109.8
C7—C6—C5	121.3 (2)	N2—C15—H15B	109.8
C7—C6—H6A	119.4	C14—C15—H15B	109.8
C5—C6—H6A	119.4	H15A—C15—H15B	108.3
C6—C7—N1	122.4 (3)	N1—C16—H16A	109.5
C6—C7—C8	117.7 (2)	N1—C16—H16B	109.5
N1—C7—C8	119.9 (2)	H16A—C16—H16B	109.5
C9—C8—C7	121.9 (3)	N1—C16—H16C	109.5
C9—C8—H8A	119.1	H16A—C16—H16C	109.5
C7—C8—H8A	119.1	H16B—C16—H16C	109.5
C8—C9—C10	121.3 (3)	N1—C17—H17A	109.5
C8—C9—H9A	119.4	N1—C17—H17B	109.5
C10—C9—H9A	119.4	H17A—C17—H17B	109.5
C9—C10—C5	117.6 (2)	N1—C17—H17C	109.5
C9—C10—C1	122.8 (3)	H17A—C17—H17C	109.5
C5—C10—C1	119.6 (2)	H17B—C17—H17C	109.5
O2—C11—N2	118.7 (3)		
			
C10—C1—C2—C3	−0.7 (4)	C6—C5—C10—C9	−2.2 (3)
C10—C1—C2—C11	−172.5 (2)	C4—C5—C10—C9	−179.9 (2)
C1—C2—C3—C4	1.8 (4)	C6—C5—C10—C1	176.7 (2)
C11—C2—C3—C4	173.8 (2)	C4—C5—C10—C1	−1.0 (3)
C2—C3—C4—O1	179.2 (2)	C2—C1—C10—C9	179.2 (2)
C2—C3—C4—C5	−2.4 (4)	C2—C1—C10—C5	0.4 (4)
O1—C4—C5—C6	2.9 (4)	C15—N2—C11—O2	−6.4 (4)
C3—C4—C5—C6	−175.7 (2)	C12—N2—C11—O2	154.2 (3)
O1—C4—C5—C10	−179.5 (2)	C15—N2—C11—C2	170.5 (3)
C3—C4—C5—C10	2.0 (4)	C12—N2—C11—C2	−28.8 (4)
C10—C5—C6—C7	−0.8 (3)	C1—C2—C11—O2	138.2 (3)
C4—C5—C6—C7	176.9 (2)	C3—C2—C11—O2	−33.7 (4)
C5—C6—C7—N1	−179.1 (2)	C1—C2—C11—N2	−38.7 (4)
C5—C6—C7—C8	3.4 (4)	C3—C2—C11—N2	149.4 (3)
C17—N1—C7—C6	−1.3 (4)	C11—N2—C12—C13	144.1 (3)
C16—N1—C7—C6	144.3 (3)	C15—N2—C12—C13	−54.0 (3)
C17—N1—C7—C8	176.1 (3)	C14—O3—C13—C12	−57.6 (3)
C16—N1—C7—C8	−38.3 (4)	N2—C12—C13—O3	55.1 (3)
C6—C7—C8—C9	−3.1 (4)	C13—O3—C14—C15	58.8 (3)
N1—C7—C8—C9	179.3 (3)	C11—N2—C15—C14	−141.8 (3)
C7—C8—C9—C10	0.2 (4)	C12—N2—C15—C14	54.6 (3)
C8—C9—C10—C5	2.5 (4)	O3—C14—C15—N2	−57.5 (3)
C8—C9—C10—C1	−176.4 (3)		

### Hydrogen-bond geometry (Å, °)

**Table:**

*D*—H···*A*	*D*—H	H···*A*	*D*···*A*	*D*—H···*A*
O1—H1···O2^i^	0.82	1.82	2.631 (3)	172
C17—H17C···Cg^ii^	0.96	2.80	3.533 (2)	134

Symmetry codes: (i) −*x*+3/2, *y*, *z*−1/2; (ii) −*x*+1, −*y*+1, *z*−1/2.
